# Self-care behaviors, medication adherence status, and associated factors among elderly individuals with type 2 diabetes

**DOI:** 10.1038/s41598-024-70000-w

**Published:** 2024-08-18

**Authors:** Mohammad Amerzadeh, Zahra Shafiei Kisomi, Mojtaba Senmar, Marzieh Khatooni, Zahra Hosseinkhani, Mahdie Bahrami

**Affiliations:** 1 Metabolic Diseases Research Center, Research Institute for Prevention of Non-Communicable Diseases, Qazvin University of Medical Sciences, Qazvin, Iran; 2 Social Determinants of Health Research Center, Research Institute for Prevention of Non Communicable Diseases, Qazvin University of Medical Science, Qazvin, Iran

**Keywords:** Self-care, Medication adherence, Type 2 diabetes, Elderly, Iran, Endocrinology, Health care

## Abstract

Elderly individuals face an increased likelihood of developing chronic diseases such as diabetes. Self-care practices and medication adherence play crucial roles in preventing complications and adverse effects of this condition. Therefore, this study aimed to determine self-care behaviors, medication adherence status, and related factors among elderly patients with type 2 diabetes. This descriptive-analytical study was conducted on 374 elderly patients with type 2 diabetes who visited educational healthcare centers in Qazvin, Iran, during 2023 (March–September). Sampling was performed using the convenience method. Data collection instruments included a demographic characteristics checklist, the summary of diabetes self-care activities questionnaire, and the Morisky medication adherence scale. Data analysis was conducted using SPSS-22 software, employing the Kolmogorov–Smirnov test, mean, standard deviation, univariate and multivariate regression analyses. The significance level was set at *p* ≤ 0.05. The mean age of participants was 67.56 ± 5.93 years. In the self-care questionnaire, the highest score pertained to adherence to the diet recommended by the treating physician (3.16 ± 1.87). In contrast, the lowest scores were related to the frequency of checking inside shoes (0.17 ± 0.93) and foot examination (0.31 ± 1.07), respectively. Furthermore, results in self-care behaviors indicated that with increasing education levels, self-monitoring of blood glucose (SMBG) significantly decreased (*P* = 0.048). This variable was considerably higher in rural residents than in urban dwellers (*P* = 0.016). Additionally, the frequency of blood glucose measurements was significantly higher in urban residents than in rural inhabitants (*p* = 0.006). Based on the results, the mean score for medication adherence among patients was 5.53 ± 1.65. Based on our findings, the level of self-care in physical activity, SMBG, and foot care among the elderly is below average. Furthermore, medication adherence in these patients is poor. We expect that managers and policymakers take steps to reduce complications and improve these two variables by developing educational programs on self-care and emphasizing the importance of treatment adherence for these patients.

## Introduction

The global population is rapidly aging, with the number individuals aged 60 and above increasing. Currently, older adults make up 12% of the world's population. According to the latest report by the World Health Organization (WHO), between 2015 and 2050, the proportion of the global population aged 60 and above is projected to nearly double from 12 to 22%^1^. In Iran, according to the latest results of the census conducted by the Statistical Center in 2017, the population of individuals aged 60 and above was approximately 7.4 million, accounting for about 9.28% of the population^2^. A crucial point to consider is that with advancing age, the likelihood of developing chronic diseases and their associated complications increases significantly^3,4^.

Diabetes mellitus (DM) is one of the most common chronic diseases and is becoming a global epidemic due to increased life expectancy, urbanization, the prevalence of obesity, and overall lifestyle changes^5^. It is estimated that by 2045, approximately 625 million individuals worldwide will be affected by diabetes^6^. Globally, type 2 diabetes mellitus (T2DM) accounts for over 90% of diabetes cases^7^ . Iran is one of the countries in the region with the highest number of individuals affected by diabetes^8^ . The prevalence of diabetes among the elderly in Iran has been estimated to be at least 14% in various studies^9,10^. Results from another study conducted in Iran have demonstrated that 33.6% of the elderly population is affected by diabetes^11^.

The increasing prevalence of diabetes imposes substantial costs on individuals, families, communities, and the healthcare system^12,13^ while reducing the quality of life^14^. Diabetes is a common functional impairment among the elderly, which affects their physical, psychological, and social functioning^14^ and leads to a decline in their health^15^. The ultimate goal in managing this disease, which is a contributing factor in 88 percent of deaths among those affected, is to achieve optimal blood glucose control and prevent complications^16^. However, only a small portion of chronic diseases, such as diabetes, are treated by healthcare professionals, and the vast majority of these diseases are managed and controlled by the individuals themselves and their families^17^. This self-management and control necessitate that patients undertake various interventions and care practices^18^. One of the disease-related behaviors that predict successful treatment and reduce negative consequences and disease severity is patient adherence to the therapeutic regimen^19^.

Adherence to therapeutic regimens and prescribed medications is a crucial factor in achieving better control of diabetes, preventing mortality, and reducing its prevalence^20^. Adherence is the extent to which an individual's behavior, including medication intake, dietary adherence, or implementing lifestyle changes, aligns with the recommendations provided by healthcare professionals^19^. Several studies have shown that medication adherence among elderly patients with diabetes is suboptimal and should be periodically and repeatedly evaluated, with the identification of associated factors^21^. Studies conducted in Uganda^22^, Ethiopia^23^, and the United Arab Emirates^24^ have reported medication adherence rates of 83.3%, 85.1%, and 84%, respectively. In contrast, studies in Switzerland and Botswana have reported low adherence rates of 40% and 52%, respectively^18,25^.

Khayali et al. reported moderate adherence to treatment among elderly patients with diabetes in Iran^20^. In the study by Sanati et al., treatment adherence was poor among the elderly^26^. Although the range of treatment adherence in patients with diabetes has been stated to be 31–71%^20^, poor adherence or non-adherence to treatment is one of the main reasons for treatment failure, increased disease complications, prolonged treatment duration, and increased healthcare costs. Therefore, sequential assessment of adherence issues and a focus on improving medication adherence in patients with type 2 diabetes are considered essential for reducing complications^27^. Another characteristic that decreases in elderly diabetic patients is self-care behaviors^28^.

Self-care is an important aspect of self-efficacy, influencing an individual's effort to perform a target behavior or behaviors and determining their ability to sustain the behavior despite potential obstacles or setbacks^29^. Self-care activities, which include monitoring blood glucose levels, following a low-fat diet, engaging in daily exercise, and checking one's feet^30^, can serve as a crucial component in the diabetes care process, improving quality of life and preventing acute and chronic complications^29,30^. Consequently, self-care assessment is one of the best methods for managing chronic diseases like diabetes^31^. However, in the study by Sadeghi et al. in Iran, the self-care confidence among elderly diabetic patients was moderate to low^29^. Similarly, in the study by Borhaninezhad et al., self-care ability in elderly diabetic patients was reported as poor^32^.

In summary, there is an ongoing need to assess the level of adherence to medication and self-care activities, and the factors associated with non-adherence to medication and self-care among individuals with T2DM^33^. Although some studies have reported poor adherence to self-care behaviors^7,34,35^ and low medication adherence in patients^12,22,36^, there is a lack of studies focusing on elderly diabetic patients. Therefore, to identify strengths and weaknesses in these two variables, this study aimed to investigate self-care behaviors, treatment adherence status, and related factors among elderly individuals with T2DM.

## Methods

### Study design

This descriptive-analytical study was conducted among elderly diabetic patients visiting educational hospitals (Velayat and Bou Ali) in Qazvin, Iran, during 2023 (March–September). The sample size in this study was calculated to be 374 individuals, considering d = 1.3, δ1 = 6.01, δ2 = 6.86, α = 0.05, and β = 0.80, using the following formula. With the addition of a 10% dropout rate, 412 individuals were included in the study^30^.

$$n = \frac{{(z_{1 - \alpha /2} + z_{1 - \beta } )^{2} [p_{1} (1 - p_{1} ) + p_{2} (1 - p_{2} )]}}{{d^{*2} }}$$

### Data collection

All individuals who met the inclusion criteria were invited to participate in the study after receiving a comprehensive explanation of the research objectives. Written informed consent forms were obtained from those who agreed to participate. The inclusion criteria for this study were: being aged 60 years or older, having received a diagnosis of diabetes for at least one year, and being under treatment with antidiabetic medications for at least six months prior to the study. The exclusion criteria included patient unwillingness to participate in the research follow-up or patient mortality.

After obtaining written consent from the patients, the questionnaires were completed through interviews conducted by the researcher. The study population consisted of patients with T2DM visiting Velayat and Bouali educational hospitals in Qazvin. A systematic random sampling method was used, where the list of controlled diabetic patients was extracted from each center, and the number of patients from each center was determined proportionally to the desired sample size. Then, patients were systematically selected from the center's list using a random number generator.

To collect the data, a three-part questionnaire was used:

*Part 1* A demographic questionnaire consisting of questions such as age, gender, education level, employment status, marital status, economic status, place of residence, underlying diseases, tobacco use, family history of diabetes, type of treatment, and diabetes-related complications.

*Part 2* A diabetes self-care activities questionnaire was used, which is a self-reporting scale composed of 15 items. These items include 10 items related to the measurement of four domains of diabetes self-management, namely diet, exercise, blood glucose testing, and foot care, and two items regarding smoking. Participants were asked to respond to these items based on their self-care activities in the past seven days. The responses were scored on an ordinal scale ranging from 0 to 7. The average score for each of the five domains was calculated, with higher scores indicating better diabetes self-care. The reliability and test–retest reliability have been demonstrated in seven studies with normative data^37^, showing sufficient reliability and validity with moderate reliability (r = 0.59–0.74)^38^. This tool has been previously validated and widely used in diabetes management research in various settings^7,16,30,38^.

*Part 3* The Morisky medication adherence scale (MMAS), developed by Morisky et al. in 2006, was used. It consists of 8 items. The MMAS questionnaire uses seven binary response options (Yes = 0, No = 1) and one four-point response option (Always = 3, Sometimes = 2, Rarely = 1, Never = 0) for scoring. A score of 6 or higher indicates good adherence to treatment. Questions 1 to 7 on this scale are scored based on the number of "no" responses (0), while questions 8 is scored separately. Scores below 6 indicate poor adherence; scores of 6 and 7 indicate moderate adherence; and a score of 8 indicates high adherence. The score range for this questionnaire is from 0 to 10. The Cronbach's alpha coefficient was calculated to be 77%^39^. In a study conducted by Negarandeh et al. in 2013, the scale was translated from English to Persian, and its validity and reliability were confirmed with a coefficient alpha of 0.72 on a sample of 204 participants^40^. Additionally, Mahramzad et al. have also translated this tool into Persian and confirmed the validity and reliability of the Persian version^41^. This questionnaire has been previously translated into Persian and implemented for diabetic patients in previous studies, and its psychometric properties have been examined^42–44^.

### Data analysis

After entering the data into the software and cleaning it, the normality of the data was assessed using the Kolmogorov–Smirnov test. If the data were normally distributed, quantitative variables were described using means (standard deviations), and qualitative variables were described using frequencies (percentages). If the data were not normally distributed, medians and quartiles were used instead. To examine the relationships between variables, both univariate regression tests and multivariable regression analyses were conducted to control for confounding variables. A significance level of *p* < 0.05 was used to indicate statistical significance. SPSS version 22 software was used for data analysis.

### Ethical approval and consent to participate

All protocols were approved by the Ethical Committee of the Qazvin University of Medical Sciences (Ethics code IR.QUMS.REC.1401.371). All methods were carried out in accordance with relevant guidelines and regulation. We provided the participants or their legal guardian(s) with an information sheet, reassured them about anonymity, freedom to withdraw and confidentiality, explained the purpose of the study and obtained their informed consent form".

## Results

Out of the total 412 patients participating in the study, 204 (49.5%) were female, and the rest were male. The mean age of the participants was 67.56 ± 5.93 years (Table 1).

**Table 1:** Demographic and clinical characteristics in older adults with T2DM in Qazvin, Iran, 2022 (n = 412).

Variables	Mean(SD) N(%)		Variables	Mean(SD) N(%)	
Gender	Female	204(49.5)	Medication adherence status	Poor	299(72.6)
	Male	208(50.5)		Medium	103(25.00)
Occupation	Employed	47(11.4)		High	10(2.40)
	Housewife	182(44.2)	Place of residence	City	203(49.3)
	Retired	179(43.4)		Village	209(50.7)
	Unemployed	4(0.1)	Underlying diseases	Yes	220(53.4)
Education level	Illiterate	79(19.2)		No	192(46.6)
	High school	145(35.2)	Family history of diabetes	Yes	181(43.9)
	Diploma	146(35.4)		No	231(56.1)
	Academic	42(10.2)	Type of treatment	Oral	300(72.8)
Marital status	Married	409(99.3)		Injection	82(19.9)
	Single	3(0.7)		Life-style	30(7.3)
Economic status	Poor	134(32.5)	Diabetes-related complications	Yes	85(20.6)
	Medium	222(53.9)		No	326(79.1)
	Rich	56(13.6)		Missing	1(0.2)

Table 2 shows the mean and standard deviation of self-care categorized by non-adherence, at least 3 times per week, and daily self-care for patients in different areas such as diet, physical activity, blood glucose monitoring, and foot care. The highest self-care was related to following the diet recommended by the treating physician 3.16 (1.87). The lowest self-care was the number of times checking inside the shoes 0.17 (0.93) and the number of times checking the feet was 0.31 (1.07). Based on the information in the table above, 85.9% of patients never participated in exercise sessions (swimming, walking, and cycling), and 93.9% of patients never checked inside their shoes. Further, 333 (80.82%) of patients consumed high-fat foods at least three times a week and 297 (72.09%) consumed high-carbohydrate foods at least three times a week. Only 6 (1.5%) of patients checked their feet daily, and about 74 (18.00%) of patients reported smoking.

**Table 2:** Frequency of adhering to self-care behavior in patients with T2DM in Qazvin, Iran, 2022 (n = 412).

Variables					
Self-care behaviours (0 to 7 days)		Mean(SD)	No self-care behavior	Self-care behavior at least 3 times in week	Self-care behavior at least daily
Diet					
Adherence to a recommended healthy diet by the treating physician		3.16(1.87)	26(6.3)	256(62.13)	46(11.2)
Adherence to a specific diet plan		2.48(2.25)	84(20.4)	226(54.85)	53(12.9)
Consumption of 5 or more servings of fruits and vegetables		2.64(1.67)	12(2.9)	323(78.40)	27(6.6)
Consumption of high-fat foods		1.83(1.43)	36(8.7)	333(80.82)	11(2.7)
Consumption of excessive carbohydrates		2.87(1.74)	8(1.9)	297(72.09)	31(7.5)
Exercise					
Engaging in at least 30 min of physical activity		1.28(1.66)	170(41.3)	209(50.73)	13(3.2)
Participation in specialized exercise sessions (such as swimming, walking, cycling)		0.59(1.58)	354(85.9)	47(11.41)	11(2.7)
Self-monitoring of blood glucose (SMBG)					
Frequency of blood glucose measurements		1.97(2.06)	88(21.4)	267(64.80)	45(10.90)
Frequency of blood glucose measurements as recommended by the healthcare team and guidelines		1.96(2.04)	87(21.1)	267(64.80)	43(10.40)
Frequency of insulin or anti-diabetic medication administration		2.94(2.27)	51(12.4)	263(63.83)	81(19.70)
Foot care					
Frequency of foot examination or inspection		0.31(1.07)	350(85.00)	51(12.39)	6(1.5)
Frequency of checking inside shoes		0.17(0.93)	387(93.9)	17(4.1)	6(1.5)
Frequency of washing feet		2.89(1.59)	27(6.6)	300(412)	31(7.5)
Frequency of drying between toes		2.7(1.72)	54(13.1)	276(66.99)	29(7.00)
Cigarette smoking	Never	241(58.5)			
	Sometimes	97(23.5)			
	Usually	74(18.00)			

Table 3 shows the results of multivariate analysis and factors associated with different domains of self-care. The relationship of all variables was analyzed separately for each self-care domain, and the significant relationships between demographic and clinical variables are shown. As the level of education increased, SMBG significantly decreased (*P* = 0.048). In rural areas, SMBG was significantly higher than urban areas (*P* = 0.016). Among the demographic and clinical variables, only age had a significant relationship with diet (*p* = 0.04). As age increased, adherence to the dietary regimen improved, and those without a family history of diabetes were less likely to follow the recommended diabetic diet (*p* = 0.008). Individuals with higher educational levels consumed foods with higher fat content (*p* = 0.011). Those without a family history of diabetes participated significantly less in exercise sessions than those with a family history of diabetes (p = 0.04). The frequency of blood glucose monitoring was significantly higher among individuals living in urban areas than those living in rural areas (*p* = 0.006).

**Table 3:** Associated factors with patients frequency of adhering to self-care behaviors.

Variables	β_coefficient	Standard error of β	*p*-value	Confidence interval
SMBG				
Education level	− 0.51	0.26	0.048	− 1.01, − 0.005
Place of residence	1.11	0.46	0.016	0.21, 2.02
Diet totally				
Age	0.13	0.07	0.044	0.004, 0.26
Adherence to the recommended healthy diet by the treating physician				
Age	0.03	0.01	0.048	0.000, 0.06
Occupation	0.29	0.13	0.028	0.03, 0.55
History of family diabetes	− 0.49	0.18	0.008	− 0.85, − 0.13
Diabetes-related complications	0.55	0.22	0.015	0.99
Consumption of high-fat foods				
Education level	0.20	0.08	0.011	0.04, 0.35
Consumption of excessive carbohydrates				
Occupation	− 0.25	0.13	0.044	− 0.050, − 0.007
Engaging in at least 30 min of physical activity				
Age	0.03	0.01	0.041	0.0008, 0.05
Type of treatment	0.28	0.13	0.038	0.01, 0.54
Participation in specialized exercise sessions (such as swimming, walking, cycling)				
Family history of diabetes	− 0.32	0.16	0.04	− 0.63, − 0.01
Frequency of blood glucose measurements				
Place of residence	0.56	0.20	0.006	0.16, 0.95
Frequency of blood glucose measurements as recommended by the healthcare team and guidelines				
Place of residence	0.55	0.20	0.006	0.16, 0.94
Frequency of insulin or anti-diabetic medication administration				
Education level	− 0.27	0.12	0.030	− 0.51, − 0.03
Economic status	− 0.36	0.17	0.035	− 0.69, − 0.03
Frequency of foot examination or inspection				
Diabetes-related complications	0.28	0.13	0.031	0.03. 0.54
Frequency of foot washing				
Family history of diabetes	0.33	0.16	0.039	0.02, 0.64

## Discussion

One of the most important aspects of diabetes treatment is the medication regimen and self-care behaviors^45^. In diseases and disorders such as diabetes, treatment and management heavily rely on patient actions through self-care^7^. Self-care behavior is a key concept in promoting health and refers to the decisions and activities an individual undertakes to cope with a health problem or improve their own health^45^.

Self-care plays a crucial role in determining a positive clinical trajectory for patients with diabetes, and most healthcare providers consider diabetes as a type of chronic disease where patients must take responsibility for self-care. The majority of studies conducted in self-care have focused on examining dietary regimen, physical activity, and medication adherence in patients with diabetes^35^.

The results of the present study indicate that the self-care status of the patients was not satisfactory in most domains. Similarly, in a study conducted by Parham et al., self-care was at a moderate level in the majority of diabetic patients^45^. The findings of the current study are consistent with the results of Asian and African studies that have reported a low level of self-care^46,47^.

This issue requires comprehensive, effective, and efficient health interventions. Perhaps for this reason, according to the WHO and the International Diabetes Federation, diabetes has emerged as a challenge in primary healthcare in the twenty-first century, and this challenge is more serious in the Middle East. However, some studies have reported conflicting results, suggesting that these differences in findings may be due to variations in the study populations and the types of tools used^48,49^.

In this study, a significant number of patients did not participate in exercise sessions, and the average self-care score in physical activity was low. These findings are consistent with other studies showing that individuals with diabetes have very low levels of physical activity^35,50^. Generally, it can be said that individuals with diabetes often have poor physical activity due to physical problems, illnesses, and disabilities, which can lead to reduced social engagement and increased dependency. However, the results of the study by Ipakchi Pour et al. showed that elderly individuals with diabetes had higher scores in physical activity compared to other domains^21^. Although this study used a different instrument to assess physical activity, the difference in results could be attributed to the level of education or awareness among individuals. Presumably, we witness this discrepancy due to cultural and educational differences between the context of this study and our study. In the study by Nelson et al., 73% of patients had appropriate physical activity^51^, which is somewhat inconsistent with our findings. One possible reason for this difference could be attributed to the research population. In Nelson et al.'s study, individuals aged 30 and above were included, whereas our study was conducted among the elderly. It is plausible that the changes associated with aging itself could influence individuals' physical activity levels. The discrepancy in results may stem from the differences in the study populations' age groups.

The results of this study indicate that age has a significant and positive correlation with adherence to dietary regimens, with older patients showing higher adherence to dietary recommendations for diabetes. This finding is consistent with previous studies that have also found that older individuals have better self-care behaviors, including dietary adherence^52^. However, the results of some studies contradict the findings of the present study. In a study by Kasan et al., older individuals had lower self-care behaviors^7^. One potential reason for the difference in results between the two studies could be the age groups of the samples. In Kassan et al.'s study, approximately 70% of the samples were under 60 years old, whereas our study involved elderly participants. It is possible that younger individuals, due to fewer limitations in their lives, place less importance on adhering to dietary regimens. On the other hand, elderly diabetic patients, apart from their disease, also face physical changes associated with aging. For this reason, the likelihood of adhering to a dietary regimen in this age group may have increased with age.

In the present study, the level of SMBG significantly decreased with higher levels of education, and individuals with higher education consumed foods with higher fat content. This finding contradicts the results of a study by Asghari et al., which found a significant positive relationship between literacy, education, and self-care improvement in patients with diabetes^53^. This discrepancy may be due to the low number of individuals with university education in the sample population of the current study. The study by Babazadeh et al. showed that the highest self-care score and the lowest hemoglobin A1c levels were observed among patients with an educational level of middle school or higher compared to those who were illiterate or had only elementary education^54^. In other words, the higher the educational level, the better the blood glucose control and the higher the self-care level, which is somewhat inconsistent with our findings. In the current study, an increase in education was associated with a decrease in self-monitoring of blood glucose. Although hemoglobin A1C was not assessed in the present study, to explain the reasons for the discrepancy, the current study was conducted among the elderly, whereas that study included all age groups above 30 years old. One potential reason for the difference in results between the two studies could be the age groups. It is more likely that the educational level is higher in age groups above 30 years, which could partly explain the discrepancy in the results.

Additionally, the results indicated that individuals without a family history of diabetes had significantly lower participation in exercise sessions compared to those with a family history of diabetes. This finding aligns with the study by Kasan et al., which also showed that having a family history of diabetes protected individuals against poor self-care behaviors^7^. This finding demonstrates that individuals with a family history of diabetes have higher sensitivity and perceived severity compared to individuals without a family history of diabetes. According to researchers, in chronic diseases such as diabetes, individuals who do not perceive the risk in their lives are more susceptible to danger. Therefore, there is a need for the development of specific programs for these individuals.

Another common complication in patients with diabetes is diabetic foot ulcers, which is a major cause of disability in diabetic patients. The results of the present study indicated that a significant number of patients had never examined their feet, and self-care in foot care was low among the patients, with only a very small number of patients examining their feet daily. These findings are consistent with other studies^55,56^. In the study by Sari et al., the standard score for foot care was low, and patients did not assess the condition of their feet^57^.

These findings may be based on the assumption that most patients believe that a daily foot examination to prevent skin injuries is not necessary. In contrast, Nelson et al. demonstrated that foot care was good among the patients, and approximately 98% of patients followed a foot care program^51^. Limited awareness of proper foot care methods among the diabetic patients in our study could be one of the major reasons for the differences in the study results.

This study showed that patients scored high in adherence to dietary regimens, contrary to the findings of Ranjbaran et al.'s study in Iran^58^, where they reported that 91% of diabetic individuals did not adhere to dietary regimens. The reason for this discrepancy can be Ranjbaran et al. different age groups, whereas the present study focused solely on the elderly population. Additionally, Ranjbaran et al. had broader inclusion criteria than the present study, which could influence the results. In the present study, overall adherence to the dietary regimen was low among the patients. However, among various aspects of self-care, the highest level of self-care was observed regarding the recommended dietary regimen by the physician. The level of adherence to the dietary regimen observed in our study was higher compared to a study conducted in Ghana, where only 6.8% of participants adhered to dietary recommendations^35^. The results of the study by Vernalli et al. were also consistent with the present study^59^. On the other hand, Eypakchi Pour et al. demonstrated that the elderly had the lowest average adherence to the dietary regimen, contrary to the findings of the present study^21^. Generally, it can be acknowledged that a healthy diet and weight reduction contribute to the management of HbA1C and reduce the risk of cardiovascular diseases. Adherence to the dietary regimen also reduces the risk of cardiovascular complications, which are responsible for a significant percentage of morbidity and mortality in diabetic patients.

One of the most important self-care behaviors is medication adherence. Low levels of medication adherence pose one of the greatest challenges for successful treatment of chronic diseases such as diabetes^60^. In the present study, the medication adherence status was poor among 72.6% of patients. Based on the overall findings, patients had poor adherence to treatment regimens. This finding indicates an unfavorable medication adherence status among the patients, which is consistent with the report of the WHO stating that medication acceptance rates among diabetic patients in developing countries are reported to be 20%^61^.

This finding is consistent with other studies in Iran and Bangladesh^60,62^. On the other hand, these findings do not confirm the results of some studies, such as Mashruteh et al.^63^ and the study by Dabaghian et al.^64^ . They reported medication adherence rates of 74.6% and 86.3%, respectively, among diabetic patients in their studies. Apart from the difference in the study population, the age groups in these two studies were different from the present study. These two studies had fundamental differences in their inclusion criteria, which could explain the discrepancy in the results. This is because the present study included elderly individuals who had been diagnosed with diabetes for at least one year and had been receiving blood glucose-lowering medications for at least six months before the study.

## Limitations

One of the main limitations of the study was the use of self-report methods, addressed by providing sufficient explanations and allowing adequate time and opportunity for participants. Another limitation was the lack of consideration for HbA1C in data collection.

Strengths: Considering the aging population trend, one of the main strengths of the study was its focus on the elderly age group. The adequate sample size allows for the generalization of results to similar cultural contexts.

Overall, the study provides valuable insights into self-care behaviors, treatment adherence, and related factors among elderly individuals with type 2 diabetes, despite some limitations. The findings can contribute to the development of interventions aimed at improving self-care and treatment adherence in this population, ultimately leading to better disease management and outcomes.

## Conclusion

The findings showed that the level of self-care in physical activity, SMBG, and foot care among the elderly was below average. Additionally, the level of treatment adherence in this group was poor. Promoting and maintaining health in diabetic patients is highly dependent on the patient's self-care behaviors, and failure to consistently engage in self-care behaviors increases the risk of short-term and long-term complications of the disease. Therefore, considering the low level of self-care among these patients, health authorities must develop a comprehensive operational plan to improve these patients' health, strengthen their self-care behaviors, and pay more attention to this issue. It is recommended that future studies employ different methods, such as observation and laboratory tests, to determine the level of self-care and treatment adherence in this age group.

## Data Availability

The datasets used and/or analyzed during the current study available from the ‎corresponding author ‎on reasonable request. The entire dataset is in Farsi language. The ‎Data can be available in English ‎language for the readers and make available from the ‎corresponding author on reasonable request.‎

## References

[1] WHO, Ageing and Health Key Facts [Internet]. Available from: https://www.who.int/news-room/fact-sheets/detail/ageing-and-health (2022).

[2] Iran SC. Selected findings of the 2016 national population and housing census. Vice Presidency Plan and Budget Organization. 2019.

[3] Pérez-Jover, V. *et al.* Inappropriate use of medication by elderly, polymedicated, or multipathological patients with chronic diseases. *Int. J. Environ. Res. Public Health* **15**(2), 310 (2018). 29439425 10.3390/ijerph15020310 PMC5858379

[4] Ashktorab, T., Baghcheghi, N., Seyedfatemi, N. & Baghestani, A. Psychometric parameters of the Persian version of the BriefCOPE among wives of patients under hemodialysis. *Med. J. Islam Repub. Iran* **31**, 20 (2017). 28955670 10.18869/mjiri.31.20 PMC5609324

[5] King, H., Aubert, R. E. & Herman, W. H. Global burden of diabetes, 1995–2025: prevalence, numerical estimates, and projections. *Diabetes Care* **21**(9), 1414–1431 (1998). 9727886 10.2337/diacare.21.9.1414

[6] Federation ID. IDF diabetes atlas 8th edition. International diabetes federation. 2017, 905–11.

[7] Kassahun, T., Gesesew, H., Mwanri, L. & Eshetie, T. Diabetes related knowledge, self-care behaviours and adherence to medications among diabetic patients in Southwest Ethiopia: a cross-sectional survey. *BMC Endocr. Disord.* **16**, 1–11 (2016). 27381349 10.1186/s12902-016-0114-x PMC4933997

[8] Ogle, G. D., Middlehurst, A. C. & Silink, M. The IDF Life for a Child Program Index of diabetes care for children and youth. *Pediatr. Diabetes* **17**(5), 374–384 (2016). 26153340 10.1111/pedi.12296

[9] Bagher, L., Farid, A., & Ozra, T. Prevalence of diabetes mellitus in Iran in 2000 (2005).

[10] Sadeghigolafshanl, M., Rejeh, N., Heravi-Karimooi, M. & Tadrisi, S. D. The effect of model-based self-management program 5A on self-efficacy of elderly patients with diabetes. *J. Diabetes Nurs.* **8**(1), 1002–1010 (2020).

[11] Mirzaei, M., Rahmaninan, M., Mirzaei, M., Nadjarzadeh, A. & Dehghani Tafti, A. A. Epidemiology of diabetes mellitus, pre-diabetes, undiagnosed and uncontrolled diabetes in Central Iran: results from Yazd health study. *BMC Public Health.* **20**(1), 166 (2020). 32013917 10.1186/s12889-020-8267-y PMC6998152

[12] Guénette, L. *et al.* Patients’ beliefs about adherence to oral antidiabetic treatment: a qualitative study. *Patient Prefer. Adher.* **9**, 413–420 (2015). 10.2147/PPA.S78628 PMC4362977 25792814

[13] Raj, G. D. *et al.* Adherence to diabetes dietary guidelines assessed using a validated questionnaire predicts glucose control in adults with type 2 diabetes. *Can. J. Diabetes* **42**(1), 78–87 (2018). 28648765 10.1016/j.jcjd.2017.04.006

[14] Fadayevatan, R., Bahrami, M., Mohamadzadeh, M. & Borhaninejad, V. Relationship of sleep quality with mental health and blood sugar control in elderly people with diabetes mellitus. *Salmand Iran. J. Ageing.* **14**(4), 380–391 (2020).

[15] Noncommunicable diseases progress monitor 2022. Geneva: World Health Organization; 2022. Licence: CC BY-NC-SA 3.0 IGO Page 225 https://www.who.int/publications/i/item/9789240047761 (2022).

[16] Elsous, A., Radwan, M., Al-Sharif, H. & Abu, M. A. Medications adherence and associated factors among patients with type 2 diabetes mellitus in the Gaza Strip Palestine. *Front. Endocrinol.* **8**, 100 (2017). 10.3389/fendo.2017.00100 PMC5465265 28649231

[17] Rwegerera, G. M. *et al.* Antidiabetic medication adherence and associated factors among patients in Botswana; implications for the future. *Alex. J. Med.* **54**(2), 103–109 (2018).

[18] Anurupa, M., Aditya, A. & Angadi, N. A study of medication adherence and self-care practices among type-2 diabetes patients in Davangere. *Natl. J. Commun. Med.* **10**(01), 12–16 (2019).

[19] Goli Roshan, A., Hosseinkhani, S. N. & Norouzadeh, R. The relationship between health literacy of elderly diabetics and adherence to treatment, Babol, Iran, 2021. *Qom Univ. Med. Sci. J.* **14**(12), 70–80 (2021).

[20] Khiyali, Z., Javidi, Z., Elham Banaei, E., Afsaneh Ghasemi, A. & Dehghan, A. Relationship between perception of aging and social support with treatment adherence in the aged with type 2 diabetes in Fasa, 2018. *J. Gerontol.* **5**(4), 54–65 (2021).

[21] Epakchipoor, F., Bastani, F. & Sabet, F. P. Self-management and medication adherence in older adults with type II diabetes referring to the endocrinology clinics of the teaching hospital affiliated to Iran University of Medical Sciences (2019). *Iran J. Nurs.* **34**(129), 1–14 (2021).

[22] Bagonza, J., Rutebemberwa, E. & Bazeyo, W. Adherence to anti diabetic medication among patients with diabetes in eastern Uganda; a cross sectional study. *BMC Health Serv. Res.* **15**(1), 1–7 (2015). 25898973 10.1186/s12913-015-0820-5 PMC4405852

[23] Abebaw, M., Messele, A., Hailu, M. & Zewdu, F. Adherence and associated factors towards antidiabetic medication among type II diabetic patients on follow-up at University of Gondar Hospital. *Northwest Ethiop. Adv. Nurs.* **2016**, 1–7 (2016).

[24] Arifulla, M., John, L. J., Sreedharan, J., Muttappallymyalil, J. & Basha, S. A. Patients’ adherence to anti-diabetic medications in a hospital at Ajman, UAE. *Malays. J Med Sci MJMS.* **21**(1), 44 (2014). 24639611 PMC3952347

[25] Huber, C.A., & Reich, O. Medication adherence in patients with diabetes mellitus: does physician drug dispensing enhance quality of care? Evidence from a large health claims database in Switzerland.* Patient Preference Adherence*. 1803–9 (2016). 10.2147/PPA.S115425 PMC5029845 27695299

[26] Sanati T, Vaezi A, Jambarsang S. Medication adherence status and its related factors among older adults in Yazd, *Elder. Health J.* **6**(2), 85–90 (2020).

[27] Acharya, A. S., Gupta, E., Prakash, A. & Singhal, N. Self-reported adherence to medication among patients with type II diabetes mellitus attending a tertiary care hospital of delhi. *J. Assoc. Physicians India.* **67**, 26–29 (2019). 31299834

[28] Esmaeilishad, B. The effectiveness of self-care training on quality of life, self-care behaviors and blood sugar in elderly without self-care behaviors. *Aging Psychol.* **6**(1), 1–11 (2020).

[29] Sadeghi, H. *et al.* The relationship between personality traits and confidence in diabetes self-care in the elderly with type 2 diabetes. *J. Health Care.* **23**(2), 133–144 (2021).

[30] Jannoo, Z. & Khan, N. M. Medication adherence and diabetes self-care activities among patients with type 2 diabetes mellitus. *Value Health Reg. Issues* **18**, 30–35 (2019). 30419448 10.1016/j.vhri.2018.06.003

[31] Association of Diabetes Care and Education Specialists, Kolb L. An effective model of diabetes care and education: the ADCES7 Self-Care Behaviors™. *Sci. Diabetes Self-Manage. Care.*** 47**(1):30–53 (2021). 10.1177/0145721720978154 34078208

[32] Borhaninejad, V., Mansouri, T., Hoseyni, R., Kojaie Bidgoli, A. & Fadayevatan, R. The relationship between diabetic knowledge and self-care among the Elderly with diabetes Type 2 in Kerman-2016. *J. Gerontol.* **1**(3), 1–10 (2017).

[33] Mata, ARd. *et al.* Quality of life of patients with Diabetes Mellitus Types 1 and 2 from a referal health centre in Minas Gerais, Brazil. *Expert Rev. Clin. Pharmacol.* **9**(5), 739–746 (2016). 26873164 10.1586/17512433.2016.1152180

[34] Kahe, M., Vameghi, R., Foroughan, M., Bakhshi, E. & Bakhtyari, V. The relationships between self-concept and self-efficacy with self-management among elderly of sanatoriums in Tehran. *Iran. J. Ageing.* **13**(1), 28–37 (2018).

[35] Mogre, V., Abanga, Z. O., Tzelepis, F., Johnson, N. A. & Paul, C. Adherence to and factors associated with self-care behaviours in type 2 diabetes patients in Ghana. *BMC Endocr. Disord.* **17**(1), 1–8 (2017). 28340613 10.1186/s12902-017-0169-3 PMC5366118

[36] Ranjbaran, S., Shojaeizadeh, D., Dehdari, T., Yaseri, M., & Shakibazadeh, E. Determinants of medication adherence among Iranian patients with type 2 diabetes: an application of health action process approach. *Heliyon* 2020;6(7). 10.1016/j.heliyon.2020.e04442 PMC7364035 32695914

[37] Toobert, D. J., Hampson, S. E. & Glasgow, R. E. The summary of diabetes self-care activities measure: results from 7 studies and a revised scale. *Diabet. Care* **23**(7), 943–950 (2000). 10.2337/diacare.23.7.943 10895844

[38] Vincent, D., McEwen, M. M. & Pasvogel, A. The validity and reliability of a Spanish version of the summary of diabetes self-care activities questionnaire. *Nurs. Res.* **57**(2), 101–106 (2008). 18347481 10.1097/01.NNR.0000313484.18670.ab

[39] Morisky, D. E., Ang, A., Krousel-Wood, M. & Ward, H. J. Predictive validity of a medication adherence measure in an outpatient setting. *J. Clin. Hypertens.* **10**(5), 348 (2008). 10.1111/j.1751-7176.2008.07572.x PMC2562622 18453793

[40] Negarandeh, R., Mahmoodi, H., Noktehdan, H., Heshmat, R. & Shakibazadeh, E. Teach back and pictorial image educational strategies on knowledge about diabetes and medication/dietary adherence among low health literate patients with type 2 diabetes. *Prim Care Diabet.* **7**(2), 111–118 (2013). 10.1016/j.pcd.2012.11.001 23195913

[41] Moharamzad, Y. *et al.* Validation of the Persian version of the 8-item Morisky medication adherence scale (MMAS-8) in Iranian hypertensive patients. *Global J. Health Sci.* **7**(4), 173 (2015). 10.5539/gjhs.v7n4p173 PMC4802120 25946926

[42] Jahangard-Rafsanjani, Z. *et al.* Effect of a community pharmacist–delivered diabetes support program for patients receiving specialty medical care: a randomized controlled trial. *The Diabetes Educ.* **41**(1), 127–135 (2015). 25420946 10.1177/0145721714559132

[43] Dehghan, M., Nayeri, N., Karimzadeh, P. & Iranmanesh, S. Psychometric properties of the Persian version of the Morisky medication adherence scale-8. *Br. J. Med. Med. Res.* **9**(9), 1–10 (2015).

[44] Ghanei Gheshlagh, R. *et al.* Determining concurrent validity of the Morisky medication adherence scale in patients with type 2 diabetes. *Iran. J. Rehabil. Res.* **1**(3), 24–32 (2015).

[45] Mahmoud, P., Ali, R., & Siamak, M. Assessment of effects of self-caring on diabetic patients in Qom diabetes association 2013. (2014).

[46] Islam, S. M. S. *et al.* Diabetes knowledge and glycemic control among patients with type 2 diabetes in Bangladesh. *SpringerPlus.* **4**, 1–7 (2015). 26101736 10.1186/s40064-015-1103-7 PMC4474969

[47] Al-Maskari, F. *et al.* Knowledge, attitude and practices of diabetic patients in the United Arab Emirates. *PloS One* **8**(1), e52857 (2013). 23341913 10.1371/journal.pone.0052857 PMC3544806

[48] Saleh, F., Mumu, S. J., Ara, F., Begum, H. A. & Ali, L. Knowledge and self-care practices regarding diabetes among newly diagnosed type 2 diabetics in Bangladesh: a cross-sectional study. *BMC public health* **12**, 1–8 (2012). 23267675 10.1186/1471-2458-12-1112 PMC3552981

[49] Gul, N. Knowledge, attitudes and practices of type 2 diabetic patients. *J. Ayub Med. College Abbottabad.* **22**(3), 128–131 (2010). 22338437

[50] Amerzadeh, M., Bahrami, M., Samie, F., Khatooni, M., Hosseinkhani, Z., Yousefi, B., & Taherkhani, O. Level of physical activity in patients with type 2 diabetes. *J. Diabetes Metab. Disord. *, 1–8 (2023).

[51] Nelson, K. M., McFarland, L. & Reiber, G. Factors influencing disease self-management among veterans with diabetes and poor glycemic control. *J. General Intern. Med.* **22**, 442–447 (2007). 10.1007/s11606-006-0053-8 PMC1829424 17372790

[52] Samuel-Hodge, C. D., Watkins, D. C., Rowell, K. L. & Hooten, E. G. Coping styles, well-being, and self-care behaviors among African Americans with type 2 diabetes. *The Diabet Educ.* **34**(3), 501–510 (2008). 10.1177/0145721708316946 PMC2668814 18535323

[53] Asghari, M., Naghibi, S. A. & Rostami, F. An investigation of the effect of training on self-care promotion in type 2 diabetic patients in noor health center in 2015. *J. Health Res Commu.* **1**(2), 22–28 (2015).

[54] Babazadeh, T., Lotfi, Y. & Ranjbaran, S. Predictors of self-care behaviors and glycemic control among patients with type 2 diabetes mellitus. *Front Public Health.* **10**, 1031655 (2022). 36711399 10.3389/fpubh.2022.1031655 PMC9874308

[55] Vafaee-Najar, A., Allahverdipour, H. & Esmaily, H. Explanation of foot care using parallel process model developedin diabetic patients. *J. North Khorasan Univ. Med. Sci.* **8**(1), 179–189 (2016).

[56] Chin, Y. F., Huang, T. T., Hsu, B. R. S., Weng, L. C. & Wang, C. C. Factors associated with foot ulcer self-management behaviours among hospitalised patients with diabetes. *J. Clin. Nurs.* **28**(11–12), 2253–2264 (2019). 30791155 10.1111/jocn.14822

[57] Sari, Y. *et al.* Foot self-care behavior and its predictors in diabetic patients in Indonesia. *BMC Res. Notes.* **13**(1), 1–6 (2020). 32005281 10.1186/s13104-020-4903-y PMC6995081

[58] Ranjbaran, S., Shojaeizadeh, D., Dehdari, T., Yaseri, M. & Shakibazadeh, E. Using health action process approach to determine diet adherence among patients with Type 2 diabetes. *J. Educ. Health Promot.* **9**, 170 (2020). 32953901 10.4103/jehp.jehp_175_20 PMC7482647

[59] Werfalli, M. M., Kalula, S. Z., Manning, K. & Levitt, N. S. Does social support effect knowledge and diabetes self-management practices in older persons with Type 2 diabetes attending primary care clinics in Cape Town, South Africa?. *PloS One.* **15**(3), e0230173 (2020). 32168342 10.1371/journal.pone.0230173 PMC7069645

[60] Khanjani Movaghar, S., Khazaei, S. & Borzouei, S. Evaluation of medication adherence and its related factors among type 2 diabetic patients. *Avicenna J. Clin. Med.* **28**(3), 158–165 (2021).

[61] Cueto M. World Health Organization. Epidemiology of diabetes mellitus 2015 updated 2016; cited 2015: Available from: www.who.int/mediacenter/factsheets/fs236/en; 2015. p. 421–4

[62] Mannan, A, *et al.* Factors associated with low adherence to medication among patients with type 2 diabetes at different healthcare facilities in southern Bangladesh. *Global Health Act.* **14**(1), 1872895 (2021). 10.1080/16549716.2021.1872895 PMC7833014 33475476

[63] Mashrouteh, M., Khanjani, N. & Gozashti, M. H. Evaluation of compliance with drug regimens in diabetic patients referred to the endocrinology clinic of Afzalipour Hospital, Kerman Iran. *Health Dev. J.* **1**(3), 182–192 (2012).

[64] Dabaghian, H., Karbaksh, M., Soheilikhah, S. & Sedaghat, M. Medication adherence in patients with type 2 diabetes referred to Imam Khomeini and Shariati Hospitals. *Payesh.* **4**(2), 103–111 (2005).

