# Author Correction: Self-care behaviors, medication adherence status, and associated factors among elderly individuals with type 2 diabetes

**DOI:** 10.1038/s41598-024-74248-0

**Published:** 2024-10-04

**Authors:** Mohammad Amerzadeh, Zahra Shafiei Kisomi, Mojtaba Senmar, Marzieh Khatooni, Zahra Hosseinkhani, Mahdie Bahrami

**Affiliations:** 1 Metabolic Diseases Research Center, Research Institute for Prevention of Non‑Communicable Diseases, Qazvin University of Medical Sciences, Qazvin, Iran; 2 Social Determinants of Health Research Center, Research Institute for Prevention of Non Communicable Diseases, Qazvin University of Medical Science, Qazvin, Iran

Correction to: *Scientific Reports*10.1038/s41598-024-70000-w, published online 18 August 2024

The original version of this Article contained an error in the spelling of the author Marzieh Khatooni, which was incorrectly given as Marzie Khatooni.

The original Article has been corrected.

